# Emotional Roles of Mono-Aminergic Neurotransmitters in Major Depressive Disorder and Anxiety Disorders

**DOI:** 10.3389/fpsyg.2018.02201

**Published:** 2018-11-21

**Authors:** Yi Liu, Jingping Zhao, Wenbin Guo

**Affiliations:** Department of Psychiatry, The Second Xiangya Hospital of Central South University, Changsha, China

**Keywords:** major depressive disorder, anxiety disorders, mono-aminergic neurotransmitters, serotonin, norepinephrine, dopamine

## Abstract

A growing body of researches support a role for dysfunction of serotoninergic, noradrenergic, and dopaminergic systems in the neurobiological processes involved in major depression disorder (MDD) and anxiety disorders (ADs). The physiological changes underlying abnormal signaling of 5-HT, NE, and DA may be due to either reduced presynaptic release of these neurotransmitters or aberrant signal transductions, and thus contributing to the alterations in regulation or function of receptors and/or impaired intracellular signal processing. Animal models demonstrate crucial responsiveness to disturbance of 5-HT, NE, and DA neurotransmissions. Postmortem and biochemical studies have shown altered concentrations of 5-HT, NE, and DA metabolites in brain regions that contribute importantly to regulation of mood and motivation in patients with MDD or ADs. Neuroimaging studies have found abnormal 5-HT, NE, and DA receptors binding and regulation in regard to receptor numbers. Medications that act on 5-HT, NE, and DA neurons or receptors, such as SSRIs and SNRIs, show efficacy in both MDD and ADs. The overlapping treatment response presumably suggests a common mechanism underlying the interaction of these disorders. In this paper, we reviewed studies from multiple disciplines to interpret the role of altered 5-HT, NE and DA mono-amine neurotransmitter functions in both MDD and ADs.

## Introduction

Major depressive disorder (MDD) is a debilitating disease characterized by depressed mood or lack of interest. Anxiety disorders (ADs) are characterized by excessive fear and anxiety. Epidemiological, cross-sectional, and prospective studies converge on the general notion that MDD and ADs are mutually interacting, and each increases the risk for the emergence and/or exacerbation of the other. MDD shares many overlapping symptoms with ADs, including attention deficit, sleep disturbance, fatigue, arousal, and psychomotor abnormality. Epidemiological, clinical, and basic studies found that 60–90% of patients with MDD simultaneously suffered from ADs (Tsuang et al., 2004). Furthermore, 50% of patients with MDD meet the diagnostic criteria for ADs at the same time, suggesting high comorbidity rate between them (Nutt, 1997; Kessler et al., 2003). Their comorbidity shows more severe symptoms and social function deficits, high suicide rate, poor prognosis, and chronic disease condition compared with those without comorbid anxiety or depressive symptoms (Devane et al., 2005; Bystritsky et al., 2008). The biological etiology of MDD and ADs is closely associated with mono-amine neurotransmitter deficiency and the abnormal function of neurotransmitter receptors. The disturbance of the three mono-amine neurotransmitter systems, serotonin (5-HT), norepinephrine (NE), and dopamine (DA) system may be present in diverse neural circuits in different brain regions (Castren, 2005; Hamon and Blier, 2013). Furthermore, the disruption of these monoamine neurotransmitters may even affect the function of their receptors and the downstream receptors. This article aimed to review the emotional roles of mono-aminergic neurotransmitters in MDD and ADs.

## The Role of 5-HT System in MDD and ADS

Disturbances in the synthesis, release, transport, and retake of 5-HT may aggravate depression. The reduction and excess of 5-HT in the brain may play an important role in the regulation of the emotional condition of the disease.

It is noted that reserpine interferes with synaptic vesicular of serotonin, depletes brain stores of 5-HT, and increases the urine concentrations of 5-HT major metabolite 5-hydroxyindoleacetic acid (5-HIAA), thereby producing depressive symptoms in human (Shore et al., 1955). These reserpine-induced depressive symptoms can be reversed by monoamine precursors (Hirschfeld, 2000). Taken together, these findings support that 5-HT systems are a biochemical basis for MDD. Coppen and associates found that lack of 5-HT in the brain may cause depressive symptoms, including depressive mood, self-accusation, and criticism (Coppen, 1967; Hogenelst et al., 2016). 5-HT deficiency in the brain may enhance negative emotions in MDD, including depressive mood, self-accusation and criticism, disgust, fear, anxiety, hostility, irritability, and loneliness (Coppen, 1967). Previous studies found that serum 5-HT concentrations in patients with MDD were significantly lower than those in healthy controls, suggesting 5-HT deficiency in patients with MDD (Bot et al., 2015; Phillips, 2017). Similarly, postmortem studies showed reduced concentrations of 5-HT and its main metabolite 5-HIAA in the brain tissue of depressed and suicidal patients (Asberg et al., 1976; Roy et al., 1989). Long-term reduction of serotonin synthesis may contribute to high susceptibility of MDD. Growing experimental and clinical evidence links the effects of antidepressants with brain 5-HT systems, and indicates that central 5-HT systems perturbance is a key factor in the pathophysiology of MDD. The serotonergic dysfunction involved in MDD development mainly includes low neuronal 5-HT synthesis and abnormal function of 5-HT receptors (Artigas, 2013). Serotonin reuptake inhibitors (SSRIs), such as sertraline, fluvoxamine, and fluoxetine, can increase extracellular 5-HT concentrations of 5-HT neurons in the midbrain dorsal raphe nucleus (Aberg-Wistedt, 1989; Artigas, 1993), and are effective in improving depressive symptoms (Fabbri et al., 2014; Castellano et al., 2016). 5-HT produces its physiological functions through the binding and interaction with multiple 5-HT receptors. Respectively, different subtypes of 5-HT receptors and 5-HT pathway can modulate different neural substrates (Wetzler et al., 1991).

Seven families of 5-HT receptors, including 5-HT1 to 5-HT7 receptors with their subtypes, have been identified (Hoyer et al., 1994; Barnes and Sharp, 1999). Numerous studies have systematically conceptualized that abnormalities of 5-HT1 (5-HT1A, 5-HT1D) and 5-HT2 (5-HT2A, 5-HT2C) receptors in the central nerve system may account for the manifestation of MDD (Hamon and Blier, 2013; Nautiyal and Hen, 2017). Postmortem and neuroimaging studies suggested that patients with MDD exhibited elevated density and/or activity of 5-HT1A autoreceptors compared with healthy controls (Parsey et al., 2006; Boldrini et al., 2008; Andrade et al., 2015), which were not sensitive to treatment of antidepressants (Lemonde et al., 2003). Autoreceptors can indirectly modulate the uptake and release of neurotransmitters, and are of importance in response to treatment (Pineyro and Blier, 1999; Ferres-Coy et al., 2013). Activation of 5-HT1A autoreceptors can lead to negative modulation of firing of serotonin system and decrease of serotonin release. However, studies show a general reduction in the density of postsynaptic 5-HT1A receptors in depressed patients, which may result in poor response to antidepressant treatment (Bhagwagar et al., 2004). Further, ligands with 5-HT1A agonist activity can produce both antidepressant (Choi et al., 2012) and anxiolytic (Vianna and Carrive, 2009) properties. Studies have also shown the aberrant sensitivity of postsynaptic 5-HT1D receptors and a distinctly higher distribution of 5-HT1D receptors in the globus pallidus in patients with MDD and/or suicide victims (Lowther et al., 1997; Whale et al., 2001; Murrough et al., 2011). Evidence indicates that 5-HT2A receptors play roles in MDD. The blockade of 5-HT2A receptors may enhance antidepressant-like profiles mediated by 5-HT1A receptors in the cortical and limbic brain regions (Artigas, 2013). Also, the antagonism of 5-HT2A receptors can induce a decreased regulation of 5-HT2A receptors that is considered to be beneficial in the treatment of MDD (Gray and Roth, 2001). Further, 5-HT2C receptor protein dysfunction has been observed in the prefrontal cortex region in suicidal patients as well (Gray and Roth, 2001). Taken together, presynaptic 5-HT1A autoreceptors show prejudicial action in antidepressant treatments, whereas the activation of postsynaptic 5-HT1A receptors in the corticolimbic networks is possibly of benefit in the antidepressant treatments. In addition, blockade of 5-HT2A receptors and 5-HT2C receptors abnormalities also play crucial roles in improving depressive symptoms.

Several important brain regions such as the prefrontal cortex, amygdala, and hippocampus are involved in the pathophysiology of MDD. The insula is also considered crucial in explaining affective deficits in depression. In a positron emission tomography study performed by Biver et al. (2018) 5-HT2 receptor specific radiotracer [I8F] altanserin was used to investigate *in vivo* distribution of 5-HT2 receptor in patients with MDD. The finding revealed that uptake of [I8F] altanserin was significantly decreased in brain regions including the right anterior insula and right posterolateral orbitofrontal cortex, indicating the diminished 5-HT2 receptor binding in these brain regions (Vicario et al., 2017; Biver et al., 2018). A meta-analysis reported reduced 5-HT1A receptor binding in the insula, raphe nuclei, hippocampus, occipital cortex, and anterior cingulate cortex in patients with MDD (Wang et al., 2016). Functional connectivity analyses revealed that individuals with MDD exhibited abnormal activity in the dorsal insula during the anticipation of painful stimuli relative to healthy controls (Strigo et al., 2013). These results provide evidence that the pathophysiology of MDD may involve serotonergic neurotransmission perturbance in various brain regions. Otherwise, serotonergic activity can affect brain activities in certain regions, which can be used to predict the severity of MDD. For example, functional neuroimaging studies observed that abnormal activities within brain regions including the insula and anterior cingulate cortex could be used to predict the severity of depressed mood (Ryan et al., 2012).

Differentially expressed multiple 5-HT receptor subtypes on multiple cell types result in 5-HT neurotransmission possessing both antianxiety and anxiogenic effects (Albert et al., 2014). The activation of 5-HT1A receptor in the hippocampal tissue can produce anxiolytic effects. Likewise, Paul’s and associates integrated evidence of anxiety from mouse genetic models involving knockout, mutation, over-expression, or suppression of genes of 5-HT1A receptors, suggesting that both pre- and post-synaptic 5-HT1A receptors contributed to anxiety phenotypes (Albert et al., 2014). High 5-HT1A autoreceptors, suppression of postsynaptic receptors or low 5-HT neurotransmission will lead to anxiety phenotype, whereas downregulation of 5-HT1A enhances anti-anxiety effects (Albert et al., 2014).

The agonism of 5-HT2A receptor in the amygdala may cause anxiety symptoms and insomnia (Bystritsky et al., 2008). 5-HT2A antagonism has been indicated to have an anxiolytic effect in subjects with generalized anxiety disorder in preclinical studies (Bressa et al., 1987). Furthermore, acute stimulation of 5-HT2A and 5-HT2C receptors projecting from the raphe nucleus to the amygdala and marginal limbic cortex may cause acute mental agitation, anxiety, and panic attack. Stimulation of 5-HT2A receptors in sleep centers of the brainstem may cause slow-wave sleep disturbances, resulting in night-time sleep arousals (Muntner, 2010). However, studies with 5-HT2C receptors agonists in elevated-plus maze model of anxiety generate conflicting results (Charney et al., 1987; Rodgers et al., 1992; Durand et al., 2003). Although 5-HT2C receptors stimulation is majorly anxiogenic, it is also anxiolytic sometimes. In the elevated-plus maze model studies, anxiolytic effects were observed though 5-HT2C receptors activation within the periaqueductal gray (Graeff, 2004). Conversely, 5-HT2C receptors agonism induced anxiogenic-like effects in the ventral part of the hippocampus and basolateral nucleus of amygdala (Alves et al., 2004; Vicente and Zangrossi, 2012). Additionally, upregulation of 5-HT2C receptors in the basolateral nucleus of amygdala could induce a similar anxiogenic effect (Li et al., 2012). Specially, the dual anxiolytic-anxiogenic effect of 5-HT2C receptors agonists may be of importance in antianxiety treatment. Clinically, in the initial treatment of SSRIs, the therapeutic effects are often delayed by the aggravation of anxiogenic-like profiles, including jitteriness or agitation (Pohl et al., 1988; Nutt and Glue, 1989). The increased anxiety induced by acute administration of SSRIs is possibly due to their predominant action on 5-HT1A autoreceptors, which may result in the reduction of 5-HT release and 5-HT neuron firing (Blier et al., 1987). Intriguingly, these acute adverse effects have been shown to be modulated by desensitization or blockade of 5-HT1A autoreceptors, as well as activation of 5-HT2C receptors (Bristow et al., 2000; Bagdy et al., 2001). Collectively, low 5-HT in central nerve system is also high risk for ADs. However, animal model of anxiety considers that high 5-HT in early postnatal phase may contribute to anxiety phenotype. 5-HT1A receptors activation is anxiolytic in chronic or long-term SSRIs treatment, whereas may produce anxiogenic-like effects due to the stimulation of presynaptic 5-HT receptors in acute SSRIs administration. 5-HT2A antagonism exerts anxiolysis, whereas 5-HT2C receptors may show region specific and dual anxiolytic-anxiogenic roles in treatment of ADs.

Several important brain regions such as the amygdala, cingulate cortex, and raphe nucleus are involved in the pathophysiology of ADs. The insular role is also crucial in the pathophysiology of ADs. In particular, SPECT and positron emission tomography studies have reported decreased 5-HT1A receptor binding in the insula, amygdala, anterior cingulate cortex, medial prefrontal cortex, and raphe nucleus in panic disorder (Neumeister et al., 2004; Nash et al., 2008). One study has reported reduced 5-HT1A receptor binding in the insula, amygdala, and anterior cingulate cortex in social anxiety disorder (Lanzenberger et al., 2007). Functional neuroimaging data provide evidence that exaggerated activation in the insular and cingulate cortices may be predictive of anxious traits (Shin and Liberzon, 2010; Vicario et al., 2017). Alvarez et al. (2015) found that anxiety-prone participants exhibited elevated activation in the bilateral dorsal anterior insula in response to anticipation of noxious event. These results suggest that the serotonergic activity has an impact on brain activity and provide evidence of altered serotonin neurotransmission in multiple brain regions or circuits in pathophysiology of ADs (Boshuisen et al., 2002).

Antidepressant drugs enhance 5-HT neurotransmission in patients with MDD.It can also improve many types of ADs, including panic disorder and generalized anxiety disorder (GAD) (Kahn et al., 1988).The decline of 5-HT neurotransmission function can not only affect the development of emotional disorders such as MDD and ADs, but can also induce emotional disturbance through activities relative to other neurotransmitter systems (Sumner et al., 2014). In general, low 5-HT has been shown to be associated with the presence of depressive and anxiety symptoms. Additionally, both 5-HT1A and 5-HT2A/2-HT2C receptors are majorly involved in depression and anxiety profiles. These observations clearly support that anxiety and depressive symptoms can be treated simultaneously.

## The Role of NE in MDD and ADS

NE is ingested into the noradrenergic nerve endings by tyrosine transporter through a precursor formation of tyrosine and NE and is converted to NE by a series of transformations. In 1979, Zis and associates proposed the NE hypothesis of MDD that depressive symptoms were caused by the decrease of NE in the central nervous system (Zis and Goodwin, 1979). Postmortem studies of depressed patients have found increased conformation of central α2-adrenergic autoreceptors and decreased NE transporter binding affinity in the locus ceruleus (Klimek et al., 1997) with no significant alteration in density of α2-adrenergic receptors in raphe nuclei (Escriba et al., 2004). Studies have observed increased binding of agonist ligands at α2-adrenergic autoreceptors on the NE neurons cell body, indicating higher functions of these NE autoreceptors and thus suggesting a lower noradrenergic neurotransmission in MDD (Hamon and Blier, 2013). The α2-adrenergic autoreceptors occur presynaptically on noradrenergic and serotonergic neuronal terminals and exert suppression effects in the release of neurotransmitters upon stimulation. In addition, postmortem studies have demonstrated elevations in mRNA levels for α2-adrenergic autoreceptors in the frontal cortex from suicide subjects, and the majority of the subjects had MDD diagnosis before death (Vicente and Zangrossi, 2012). Possible interpretation of these detections is that hypersensitive presynaptic α2-adrenergic autoreceptors can contribute to reduction in NE and serotonin release. NE reduction in the central nervous system is associated with depletion of positively affective resources in patients with MDD, including decrease in pleasure, interest, happiness, alertness, energy, and passion and loss of confidence. Patients with MDD had lower NE function in lobar NE, causing anhedonia, loss of energy and passion, and other relative depressive symptoms (Bystritsky et al., 2008). Conversely, the symptoms of anxiety were assumed to be caused by hyperactivity of NE in the central nervous system. In stress conditions, corticotropin-releasing factor can activate the NE energy pathway in the locus coeruleus-temporal hippocampal, which releases NE and inducing wakefulness and anxiety symptoms (Muntner, 2010). Further, researchers observed elevated serum catecholamine concentrations in patients with GAD, indicating the excess of NE (Homan et al., 2015; Reader et al., 2015). Preliminary evidence suggests that single-nucleotide polymorphism involved in the function of adrenergic receptors is a susceptibility factor for general anxiety disorder (Zhang et al., 2017). Animal model studies found that antagonism of β-adrenergic receptors within central nerve system could attenuate the anxiogenic effects of cocaine (Wenzel et al., 2014) and disrupted anxiety-like phenotypes including aversive, fear and stress-related behaviors (Kindt et al., 2014; McCall et al., 2017).

## The Role of DA in MDD and ADS

DA is a neurotransmitter in the hypothalamus and pituitary that is a key neurobiological substrate for reward, concentration, motivation, psychomotor speed, and the ability to experience pleasure, which may play a role in the modulation of human emotions (Coppen, 1967). Dopaminergic activity has been demonstrated to be involved in depressive (Ryan et al., 2012) or anxious (Vicario et al., 2017) processing. A strong evidence links reward-related, hedonic, and motivated behaviors with the mesolimbic DA system (Cabib and Puglisi-Allegra, 1996). Impairment of these functions are all prominent characteristics of MDD. Moreover, immediate bidirectional control (inhibition or excitation) of specified midbrain DA neurons modulates multiple independent depressive symptoms caused by chronic stress, suggesting that processes affecting depressive symptoms alter the DA neural encoding of action in the limbic circuitry (Tye et al., 2013). Further, poor functioning of DA neurons may cause depressive symptoms, including hopelessness and loss of interest (Kasch et al., 2002; Dunlop and Nemeroff, 2007). Patients with MDD showed lower level of DA metabolites in the cerebrospinal fluid compared with healthy controls (Jokinen et al., 2007). Deficiency in DA receptor function may lead to the failure in inhibition from the prefrontal cortex to the amygdala, and induce the over excitability of the amygdala, resulting in the emergence of fear and pathological anxiety.

DA receptors have two subtypes, the D1 and D2 receptor. Studies have demonstrated reduced dopamine transporters density and D2 receptor binding in the striatum in patients with social anxiety disorder compared with healthy controls (Schneier et al., 2000; Shin and Liberzon, 2010).DA blockers can increase the severity of social fear symptoms (Clausius et al., 2009). Furthermore, the level of homovanillic acid, a DA metabolite in the cerebrospinal fluid, had a lower level in patients with depressive comorbid with social fear (Jokinen et al., 2007). Thus, these conclusions may suggest DA or DA receptor dysfunction in patients with MDD, ADs, and with comorbidities.

Furthermore, the activity of dopaminergic neurons in the ventral striatum could affect insular activity, which could be used to predict the symptom severity of MDD and ADs (Black et al., 2002). There was evidence that reduction in expression of dopamine receptors in the striatal pathways and enhanced functioning of the insula and adjacent operculum were involved in mood alterations and associated behaviors such as eating disorder (Stice et al., 2009; Frank et al., 2012). Animal model studies and clinical case reports also suggested dopaminergic circuit could contribute to mood regulation through the insula. The agonist of DA receptor, particularly the D3 receptor, could reduce cerebral blood flow in the insula of the baboons, which was further supported by the treatment of MDD in several clinical trials (Goldberg et al., 2004; Zarate et al., 2004).

## Interactions Between 5-HT, NE and DA

The NE and 5-HT neurotransmitter systems are mutually interacted in the central nervous system (Quesseveur et al., 2013). NE plays a role in the regulation of the release of 5-HT. Stimulating the α2 receptor on the axon terminals can inhibit the release of 5-HT, and stimulating the α1 receptors on neuronal cell bodies or dendrites may cause positive feedback on the release of 5-HT. At the same time, 5-HT systems can exert negative influence on NE systems through the 5-HT2A and 5-HT2C receptor-mediated mechanisms (Hamon and Blier, 2013). Evidence suggests that 5-HT2A receptors can enhance the release of NE under the SSRIs treatment (Sullivan et al., 2005). Both NE and 5-HT nerve fiber project signaling in the frontal cortex and hippocampus play an important regulatory role in cognition and behavior, especially in mental and emotional regulation in the central nervous system. Further, the two neurotransmitters signaling in the hippocampus and frontal cortex has been the target of a large proportion of research on MDD, ADs, and their treatments (Graeff et al., 1996).

There is multiple interaction between the serotonergic and dopaminergic systems. Increased or reduced neurotransmission of serotonergic or noradrenergic systems can affect dopamine function and induce similar changes in dopaminergic signaling. The antidepressant and anxiolytic efficacy of clinical therapeutics may partly result from the alterations in DA neurotransmission of the DA reward-learning circuit signaling. In patients with MDD or ADs, dopamine-related disturbances can be presumably improved through this mechanism. Several positron emission tomography and SPECT studies found that increase in striatal dopamine receptor binding and dopamine transporter availability correlated with improvement in Hamilton Depression Rating Scale scores (Mischoulon et al., 2002; Yang et al., 2008). In addition, substantial interaction exists between serotonergic cells of the midbrain raphe and target dopaminergic cell bodies in the ventral tegmental area in central nervous system. Findings of Mataix et al. indicated that stimulation of 5-HT1A receptors in the medial prefrontal cortex could enhance activity of the ventral tegmental area DA neurons, along with meso-cortical DA release. In particular, 5-HT systems can also exert a negative influence on DA systems through the 5-HT2A and 5-HT2C receptor-mediated mechanisms (Clausius et al., 2009). Both of them play important roles in the regulation of mood and mental movement in the central nervous system. Acute stimulation of the basal 5-HT2A receptor may inhibit DA function in the basal area and causes acute motor changes, including psychomotor retardation and dystonia. Stimulating the 5-HT2A receptor in the midbrain may inhibit DA activity, causing apathy and sex reduction. Additionally, 5-HT2C receptors play a role in the tonic regulation of ascending dopaminergic activity, which may be a potential effect of antidepressant drugs.

## Conclusion

**FIGURE 1:**
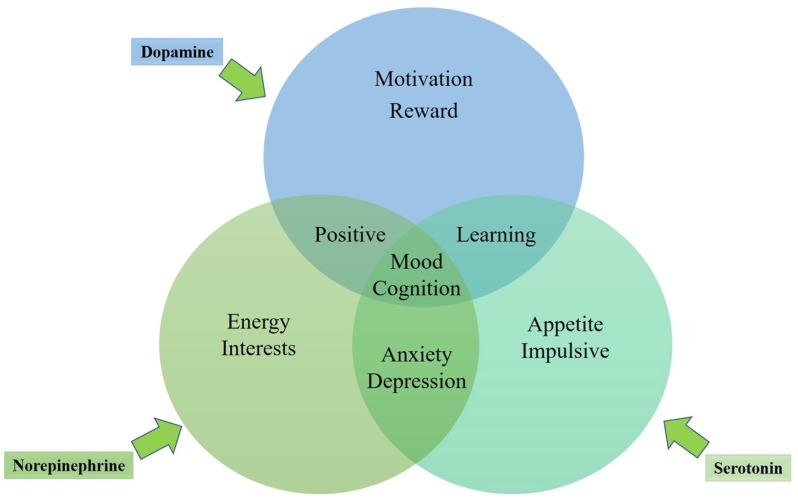
Emotional role of 5-HT, NE and DA in the regulation of depression and anxiety.

In general, the three mono-aminergic neurotransmitter systems are mutually interacting, each playing roles in the regulation of diverse human emotions (see the mechanism in Figure 1). Depression and anxiety may be directly caused by dysfunction in brain areas including hippocampus, amygdala, and the prefrontal cortex (Mayberg et al., 1997; Steffens and Krishnan, 1998; Bremner, 2002)or by the neural systems modulated by mono-amine neurotransmitter systems in these brain regions (Delgado and Moreno, 2000). The diffuse nerve fibers of 5-HT, DA, and NE in the hypothalamus, thalamus, basal forebrain, and the prefrontal cortex could play roles in the regulation of dysmnesia. However, lack of energy and fatigue could associate with hypofunction of NE and DA in the prefrontal cortex (Muntner, 2010). In other words, part of the depressive and anxiety symptoms may be related to a certain neurotransmitter system, while other symptoms may be related to a variety of other neurotransmitter systems. No single drug can fully improve any psychiatric disorder. However, a certain drug can possibly improve depressive mood by enhancing the information-processing function in a brain region, while another drug with a different mechanism can ease other symptoms such as insomnia, anxiety or concentration deficiency by improving the information-processing function in other brain regions. The treatment with the same antidepressant drug is effective for both depressive and anxiety symptoms, also supporting the possibility of the same neurobiological neurotransmitter dysfunction mechanism underlying the symptoms of MDD and ADs.

## Author Contributions

YL wrote the protocol and first draft of the manuscript. YL and JZ contributed to concept and design. YL and WG revised critically the manuscript for important intellectual content.

## Conflict of Interest Statement

The authors declare that the research was conducted in the absence of any commercial or financial relationships that could be construed as a potential conflict of interest.
